# Copper Sulfide and Graphite Felt Composites as Promising Electrode Materials for Sodium-Ion Batteries

**DOI:** 10.1021/acsami.3c18260

**Published:** 2024-03-12

**Authors:** Egle Usoviene, Egidijus Griskonis

**Affiliations:** Department of Physical and Inorganic Chemistry, Kaunas University of Technology, Radvilenu str. 19, LT-50254 Kaunas, Lithuania

**Keywords:** sodium-ion batteries, graphite felt, copper sulfides, hydrothermal synthesis, anode materials

## Abstract

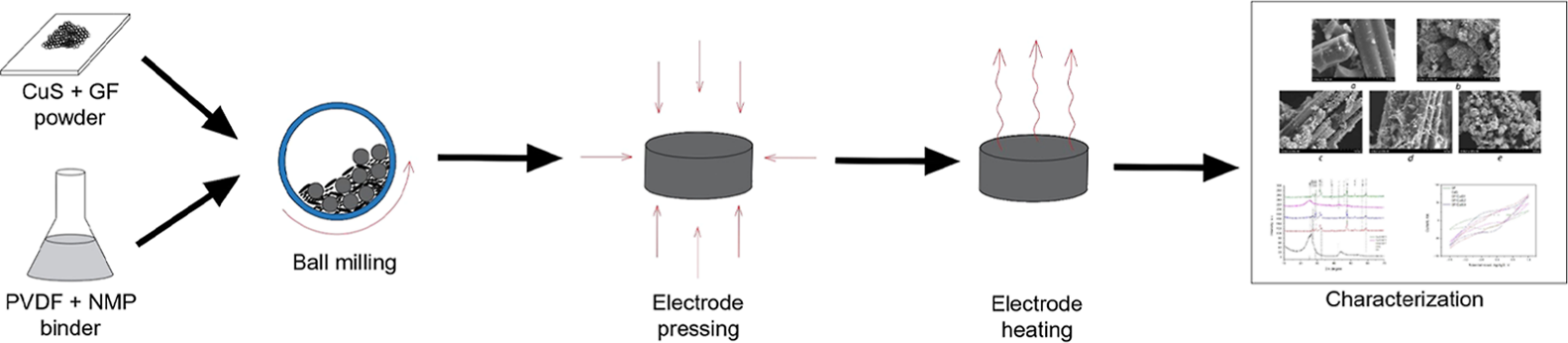

The most prominent and widely used electrical energy storage devices are lithium-ion batteries (LIBs), which in recent years have become costly and deficient. Consequently, new energy storage devices must be introduced into the current market. Sodium-ion batteries (SIBs) are starting to emerge as a promising solution because of sodium’s abundance and low cost. To offer these batteries into the current market, their properties must match surpass those of LIB predecessors, necessitating the need for research in this field. In this research work, three methods of graphite felt (GF) and copper sulfide (Cu_*x*_S) composite preparation using a hydrothermal approach have been explored and compared. The obtained samples exhibited different morphologies and thermal properties when different hydrothermal composite preparation methods were used. The areal charge capacitance values of these samples differed from 8.81 to 13.65 mF/cm^2^, and the areal discharge capacitance values differed from 10.06 to 13.65 mF/cm^2^. Notably, these achieved values are higher than those of the Cu_*x*_S and GF single substances.

## 1 Introduction

The transition from fossil fuels to renewable energy sources is needed to lessen the environmental effects caused by the emissions of greenhouse gases. This transition is attributed to the collapse of fossil fuel consumption due to its depleted reserves.^1,2^ Because of this, an energy production crisis occurred, which has created a pathway for more sustainable global development goals.^3^ Renewable energy sources used to combat this problem are mainly solar and wind.^4,5^ The excess electrical energy produced by these renewable energy sources needs to be stored and accessible at any given time because of inconsistent weather conditions, and the consumers need to use electrical energy after its production. For this reason, the supply of various electrical energy storage (EEs) devices needs to be accessible in the current market.^1,6^ There are many EEs devices that are used and are mainly lithium-based.^7,8^

EEs devices can be classified into four main groups: electrical, electrochemical, chemical, and mechanical.^9^ Primary and secondary batteries fall into the category of EEs devices. The first alkali metal-based batteries commercialized over 30 years ago were lithium-ion batteries (LIBs), which are still used by many consumers today.^10,11^ This is because LIBs exhibit the highest energy efficiency and density, and many other commercial batteries cannot match such electrical properties.^12^ The biggest problems that LIBs face are their high cost, elemental lithium and its cathode material (cobalt, nickel) deficiency, few existing recycling methods, and the negative effects on the environment caused by LIB production. The high cost of lithium is mainly impacted by the limited resource availability of this element because of the fast-growing EV market.^13^ Recycling LIBs is not an easy process because the batteries are hard to break down and they pollute the soil and underground water streams.^14^ Negative environmental effects are also caused by the purification of lithium salts in the industrial battery manufacturing process.^15^ For these reasons, in recent years, new battery technologies have started to emerge.

Battery technologies that have emerged to replace lithium-ion are sodium (Na), calcium (Ca), and magnesium (Mg)-based battery candidates.^16^ Before the initial production of the first alkali metal-based batteries, sodium-ion batteries (SIBs) were studied alongside LIB battery technologies because of sodium’s chemical/electrochemical similarities to lithium; however, LIB was thought to hold more commercial promise.^17^ This led to the reduction of interest in SIB technologies, but in recent years, they have started to emerge once again. Similarities of sodium and lithium ions include Li^+^ and Na^+^ oxidation states with one electron in their valence shell and similar standard electrode potentials, which is −2.71 V for sodium and −3.04 V for lithium .^18^ This would lead to the fact that SIBs could hold great potential to match up with existing LIB predecessors. Also, there is ongoing research on SIB electrode materials that can be implemented into large-scale applications. These electrode materials need to be low-cost, readily available, have a low negative environmental impact, and be safe to use.^19^ Materials that could be used in SIB electrode production are carbon and noncarbon compounds, oxides, sodium-based polyanions, and others.^20,21^

Transitional metal sulfides (TS_*x*_; T: Cu, Mn, Fe, etc.) have been explored as negative electrode materials for SIBs.^22^ In theory, these materials possess better theoretical specific capacity than transition metal oxides, which are widely investigated as electrode materials for SIBs. This is because when oxygen is replaced with a lower electronegative element, such as sulfur, the material’s performance increases.^23^ Copper sulfides (Cu_*x*_S) consist of various polymorphs of Cu_*x*_S, where *x* values vary from 0.5 to 2. These polymorphs have their own properties, one of which is low band gap values (1.2–2.5 eV^24^), which indicate that they can be applied in electrical devices and used as electrode materials. For this reason, Cu_*x*_S can be applied to SIB technologies. While in theory being good electrode materials for SIBs, they still encounter problems that are linked to electrochemical mass transportation, volume change, and other effects.^25^ Despite these problems, there is a wide range of positive electrode properties, which include high electronic conductivity, rich redox reactions, and high specific capacity (337–560 mAh g^–1^).^26,27^ To apply Cu_*x*_S into battery technologies, they are often coupled with carbon materials to make various negative electrode composites.

Graphite felt (GF) and carbon felt (CF) are carbon materials that can be produced in simple conversion reactions while still being electrochemically stable, chemically resistant, and electrically conductive, having a low density, and being low cost.^28,29^ They are widely used as electrode materials^30^ in vanadium redox-flow,^31^ sodium–manganese hybrid,^32^ lead-acid,^33^ and other types of batteries. When GF or CF are coupled with other materials, they can create three-dimensional electrode geometries.^34^ This is important for the accommodation of volume change in charge/discharge processes occurring while increasing the volume at the contact area of electrode/electrolyte interfaces and enhancing ion diffusion processes.^35^ While they share similarities, their electrical properties greatly differ: GF exhibits more superior electrical properties because of the unique hexagonal layer structure with highly conductive electrons, while CF is made from a variety of carbonaceous materials that are irregularly arranged, which can limit the movement of electrons.^36,37^ This means that GF can ensures an “easier” transfer of electrons, while in CF, this process is more distorted and may result in lower electrical conductivity. All of these properties must be considered when choosing between CF or GF, but because of the superior electrical properties of GF, it was chosen to be used in this work.

To research new negative electrode production technologies, three hydrothermal methods of GF and Cu_*x*_S composite anode materials were successfully explored. In this work, Cu_*x*_S synthesis is carried out using different starting materials with the addition of ball-milled GF: (1) elemental copper, elemental sulfur, and GF;^38^ (2) copper sulfate, dimethyl sulfoxide (DMSO), and GF;^39^ and (3) elemental copper, ethyl acetate, thiourea, DMSO, and GF.^40^ All three synthesized and coupled GF/Cu_*x*_S electrode samples exhibited different properties, which were analyzed using X-ray diffraction analysis (XRD), scanning electron microscopy (SEM), energy-dispersive X-ray spectroscopy (EDX), simultaneous thermogravimetric analysis (STA), voltammetric measurements (VA) of resistivity, cyclic voltammetry (CV), and open circuit voltage (OCV) analysis.

## 2 Experimental Section

### 2.1 GF Preparation

GF (SIGRACELL GFD 4,65 EA) used as an electrode material was purchased from “SGL Carbon GmbH” (Germany). GF microtweets were milled using a “Fritsch Pulverisette 9” planetary-vibrational ball miller with 10 Hz vibrational frequency for 60 s. Milled GF was used in the pressure vessel with various materials for Cu_*x*_S synthesis and its better insertion onto GF.

### 2.2 Material Synthesis

#### 2.2.1 GF/Cu_*x*_S_1

For the first GF/Cu_*x*_S_1 sample synthesis, 0.01 mol (0.64 g) elemental copper (Cu, 99.5%, “Reachem”) and 0.01 mol (0.32 g) elemental sulfur (S, a. p., “Reachem”) was used with the addition of 0.5 g of GF. Before the hydrothermal process, both Cu and S solid-state materials were ground using an agate mortar grinder and transferred to a pressure vessel with the addition of 5 mL of distilled water. Distilled water was added to the mixture to create an aqueous environment for the reaction. Synthesis of GF/Cu_*x*_S_1 was carried out for 120 min at 150 °C temperature. After completion, the pressure vessel was cooled down to room temperature naturally. The prepared GF/Cu_*x*_S_1 sample was filtered using filter paper and washed with distilled water five times before being allowed to dry naturally overnight. For comparison of the results, the Cu_*x*_S_1 sample was synthesized using the same method mentioned previously, the only difference being that no GF was added to the pressure vessel, only elemental copper, sulfur, and water. This Cu_*x*_S_1 sample was chosen as a reference because only synthesized Cu_*x*_S (*x* = 0.5–2) without any additional components would be present.

#### 2.2.2 GF/Cu_*x*_S_2

For the second GF/Cu_*x*_S_2 sample synthesis, 0.002 mol (0.23 g) copper sulfate pentahydrate (CuSO_4_·5H_2_O, a. p., “Eurochemicals”) and 5 mL of DMSO (p., “Eurochemicals”) were used. Before the hydrothermal process, CuSO_4_·5H_2_O, DMSO, and 0.5 g of GF were transferred to a pressure vessel and then heated for 360 min at 180 °C temperature. After completion, the vessel was cooled down naturally to room temperature. The prepared GF/Cu_*x*_S_2 sample was filtered using filter paper and washed with distilled water five times before being allowed to dry naturally overnight.

#### 2.2.3 GF/Cu_*x*_S_3

For the third GF/Cu_*x*_S_3 sample synthesis, 0.005 mol elemental copper (Cu, 99.5%, “Reachem”) and a 5 mL sample solution of 1 M thiourea ((NH_2_)_2_CS, 99%, “Chempur”) were used. Before the hydrothermal process, elemental copper was ground using an agate mortar grinder. To the solution, 1 mL of ethyl acetate (EtOAc, a. p., “Eurochemicals”) and 1 mL of DMSO (p., “Eurochemicals”) were added. Before the hydrothermal process, elemental copper, EtOAc, DMSO, (NH_2_)_2_CS, and 0.5 g of GF were transferred to a pressure vessel and then heated for 360 min at 180 °C temperature. After completion, the vessel was cooled down naturally to room temperature. The prepared GF/Cu_*x*_S_3 sample was filtered using filter paper and washed with distilled water five times before being allowed to dry naturally overnight.

### 2.3 Electrode Preparation

A liquid slurry was used as the binding solution required for electrode preparation. In this study, 15% PVDF/NMP binder was used: polyvinylidene difluoride (PVDF, “3M Applied Materials Division”) was dissolved in *N*-methyl-2-pyrrolidone (NMP, 100%, “VWRChemicals”) solvent overnight using a magnetic stirrer at room temperature. The prepared binder was then used with the samples in a ball miller.

First, the samples were placed in a ball miller for 2 h at 500 rpm to homogenize; after that, 10 wt % of the PVDF/NMP binder solution was added, and the ball milling process was continued for another 2 h. The prepared slurry was extracted from the ball miller and placed in a circular press lined with aluminum foil overnight. The diameter of the formed tablets was equal to 14 mm with varying thickness but not more than 2 mm. After that, the prepared electrodes were extracted and heated overnight at 100 °C in a Petri dish lined with mesh and a few droplets of distilled water to remove the NMP solvent from the electrodes. The prepared electrode samples were then used for electrochemical analysis.

### 2.4 Structural, Morphological, and Elemental Composition Analysis

Sample structural analysis was carried out using the XRD method on a “Bruker D8 ADVANCE” (Bruker Corporation, Billerica, MA, USA) machine. Analysis was performed using Cu Kα radiation with a Ni filter with a step size of 0.02° and measured intensity for 0.5 s in the range from 3.0 to 70.0° 2θ degrees. The obtained peaks were analyzed using “Search Match” computer software and identified with those of the PDF-2 database. Sample analysis results are presented in the measured intensity range of 10.0–70.0° 2θ degrees because no peaks were observed in the range from 3.0 to 10.0° 2θ degrees.

Surface morphological and elemental composition analyses were carried out using a high-resolution scanning electron microscope “Hitachi S-3400N” (Hitachi, Ltd., Tokyo, Japan) with a “Bruker X Flash Quad” (Bruker AXS GmbH, Karlsruhe, Germany) energy-dispersive X-ray (EDX) detector. Images used for SEM analysis were magnified 5000× times.

### 2.5 Thermal Analysis

Sample thermal analysis was carried out using the STA method with a “Netzsch STA 409 PC Luxx” machine under atmospheric conditions. 2 mg of the analyzed material was inserted into aluminum crucibles and heated at 10 °C per minute from 30 to 780 °C temperature.

### 2.6 Electrochemical Analysis

VA of resistivity were done using the two-piston method with a potentiostat–galvanostat “BioLogic SAS SP-150” (BioLogic, Seyssinet-Pariset, France) and its “EC-Lab v10.39” computer software. In between the two pistons, the sample powder was distributed to a specific sample height but not more than 12 mm. The top piston, with a mass of 117 g, pushed the sample powder with an area of 9.5 mm^2^. CV was deployed for three sample slope calculations in the ranges of 0.1 to −0.1 V, 0.5 to −0.5 V, and 1 to −1 V using the computer program. Analysis results were used for sample resistivity measurements, from which average sample values are taken from all three measured ranges.

Electrochemical sample analysis was carried out with CV and OCV methods using the potentiostat–galvanostat “BioLogic SAS SP-150” (BioLogic, Seyssinet-Pariset, France) with “EC-Lab v10.39” computer software. For this analysis, electrodes placed in a PTFE electrode holder with a working area of 0.785 cm^2^ were used with a 1 M Na_2_SO_4_ electrolyte. The OCV analysis results were recorded for 20 min and 30 s using a 1 mV/h scanning rate. CV results were recorded using a potential range of −1.5 to 1 V with a scanning rate of 5 mV/s. In this analysis, a three-electrode cell was employed: Pt plate was used as a counter electrode, saturated Ag/AgCl (*E* = 0.157 V vs SHE) was used as a reference electrode, and the synthesized and prepared tablet-shaped sample electrodes placed in the electrode holder were used as a working electrode.

## 3 Results and Discussion

Synthesis of Cu_*x*_S for the GF/Cu_*x*_S_1 sample when producing copper sulfide from elemental copper Cu and sulfur S is carried out using a solid-state reaction at 150 °C for 2 h. In the reaction vessel, water was added to create an aqueous environment where sulfur acts as an oxide passivating layer while allowing Cu_*x*_S to form. The reaction 1 occurring in the pressure vessel is listed below
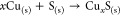
1Synthesis of Cu_*x*_S for the GF/Cu_*x*_S_2 sample using CuSO_4_·5H_2_O and DMSO occur at 180 °C in a pressure vessel for 6 h. The initial reaction that takes place is the loss of water in CuSO_4_·5H_2_O followed by the decomposition of DMSO. When DMSO decomposes, it releases methanethiol CH_3_SH, which then reacts with Cu^2+^ cations and creates copper methanethiolate CH_3_SCu, which, after some time, decomposes into Cu_*x*_S.

**Figure 1:**
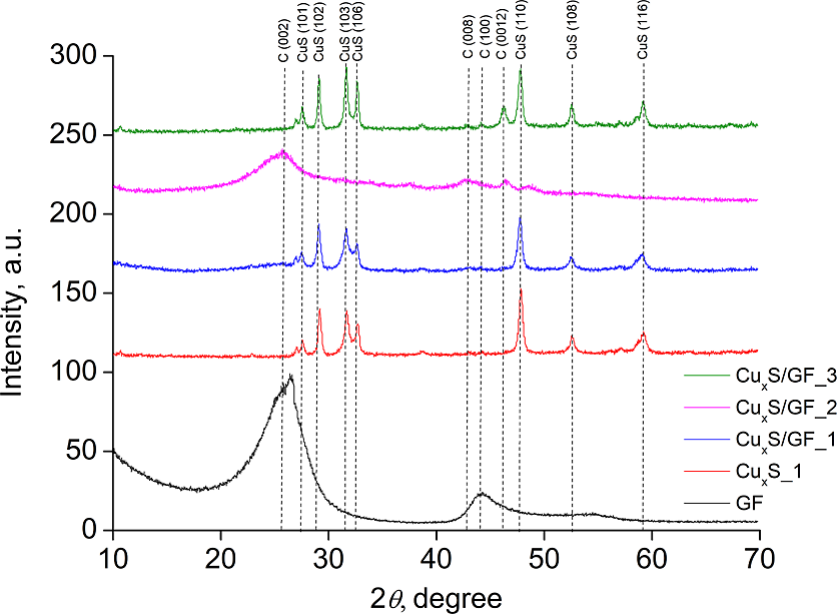
XRD patterns of Cu_*x*_S_1, GF, and GF/Cu_*x*_S_1, 2, and 3 samples synthesized using different materials.

The Cu_*x*_S used in the GF/Cu_*x*_S_3 sample was synthesized by the decomposition of thiourea at 180 °C, resulting in various chemical compounds among which hydrogen sulfide (H_2_S) was one. Elemental copper in the pressure vessel reacts with this released H_2_S gas and forms Cu_2_S. DMSO and EtOAc additives in the pressure vessel are used for the reaction with the decomposition products of thiourea to produce elemental sulfur S, which then reacts with elemental Cu. Reactions of these compounds resulting in Cu_*x*_S formations (2–4) are listed below
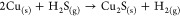
2
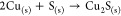
3
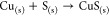
4Sample crystalline structure analysis was carried out using the XRD method. Different structural phases of GF/Cu_*x*_S_1, 2, and 3 electrode materials were investigated and were compared to CuS (PDF no. 76-1725) and graphite C (PDF no. 74-2330) peaks. Different structural phases of GF and Cu_*x*_S were observed in samples and can be seen in Figure 1.

Diffraction peaks of the GF/Cu_*x*_S_1 sample, as observed in Figure 1, indicated that the identified peaks can be attributed to the covelline CuS (PDF no. 76-1725) phase [CuS (101), CuS (102), CuS (103), CuS (106), CuS (110), CuS (108), and CuS (116) at diffraction angles of 27.46, 29.12, 31.52, 32.71, 47.85, 52.62, and 59.18°] and the graphite C (PDF no. 74-2330) phase [C (100) at a diffraction angle of 44.05°]. Diffraction peaks of the GF/Cu_*x*_S_2 sample can be attributed to the graphite C (PDF no. 74-2330) phase [C (002), C (008), C (100) and C (116) at diffraction angles of 25.79, 43.08, 44.28, and 46.18°]; however, no crystalline Cu_*x*_S phases were identified. This could mean that the amount of synthesized Cu_*x*_S in the GF/Cu_*x*_S_2 sample is much lower than that of GF. Diffraction peaks of the GF/Cu_*x*_S_3 sample indicated both covelline CuS (PDF no. 76-1725) and graphite C (PDF no. 74-2330) phases [CuS (101), CuS (102), CuS (103), CuS (106), C(008), C (100), C (0012), CuS (110), CuS (108), and CuS (116) at diffraction angles of 27.46, 29.12, 31.52, 31.52, 43.08, 44.28, 46.18, 47.85, 52.62, and 59.18°]. This means that the only sample with visible diffraction peaks of both Cu_*x*_S and GF is sample GF/Cu_*x*_S_3.

**Figure 2:**
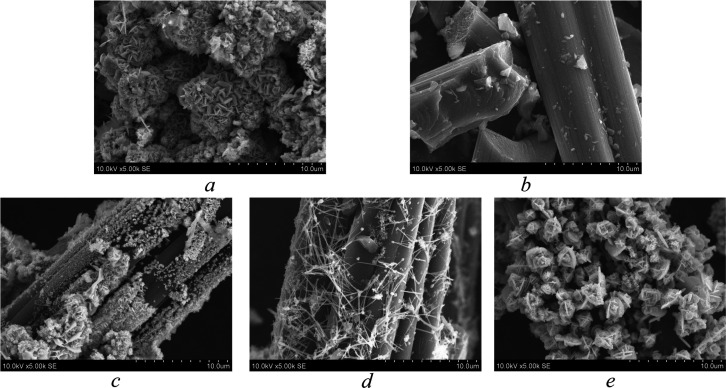
SEM images of (a) Cu_*x*_S_1, (b) GF, (c) GF/Cu_*x*_S_1, (d) GF/Cu_*x*_S_2, and (e) GF/Cu_*x*_S_3 samples.

Sample morphological analysis was carried out to observe the way in which synthesized Cu_*x*_S interacts on GF and the difference in the achieved morphologies when using different materials for Cu_*x*_S synthesis. The SEM images of Cu_*x*_S_1 (Figure 2a) and GF (Figure 2b) are presented for sample comparison. Analysis results are shown in Figure 2.

Figure 2 demonstrates SEM images showing the morphologies of reference and synthesized samples. When synthesizing Cu_*x*_S with GF in the same vessel, Cu_*x*_S effectively binds to the fibers of GF. It is clearly seen that when using different reactants for Cu_*x*_S synthesis, the crystalline structure morphologies on GF microfibers differ when analyzed by visual observation. Synthesized Cu_*x*_S in the GF/Cu_*x*_S_1 sample (Figure 2c) has smaller and scarcer particles bound to GF fibers when compared to those in the GF/Cu_*x*_S_2 (Figure 2d) and GF/Cu_*x*_S_3 (Figure 2e) samples. The GF/Cu_*x*_S_2 sample exhibits needle-like crystalline structures that resemble an interconnected cobweb that is grown on the GF microfibers. A sample that exhibits the most crystalline growth is GF/Cu_*x*_S_3 (Figure 2e). It has dense flower-like crystalline structures grown on GF microfibers that cover them up almost completely.

For sample surface elemental composition analysis, EDX was used. The elemental composition of the three samples, GF/Cu_*x*_S_1, 2, and 3, were analyzed, including carbon (C), copper (Cu), and sulfur (S). The sample composition and atomic Cu/S ratio varied when using different hydrothermal methods for Cu_*x*_S synthesis. These analysis results can be seen in Table 1. The elemental composition of individually synthesized Cu_*x*_S_1 is also presented here, which shows a very close atomic ratio of Cu and S as in the case of sample GF/Cu_*x*_S_1, when the same synthesis route was used (only without GF).

**Table 1:** Elemental Composition Dependence When Using Different Hydrothermal Methods of Cu_*x*_S Synthesis for Cu_*x*_S_1 and GF/Cu_*x*_S_1, 2, and 3 Samples

sample	atomic percentage *a*_t_, % (error, %)			atomic Cu/S ratio
	C	Cu	S	
Cu_*x*_S_1		49.6 (1.3)	50.4 (0.7)	0.98
GF/Cu_*x*_S_1	54.2 (5.0)	24.1 (1.2)	21.7 (0.4)	1.11
GF/Cu_*x*_S_2	93.8 (7.9)	4.9 (0.8)	1.3 (0.1)	3.77
GF/Cu_*x*_S_3	14.6 (0.8)	52.4 (1.2)	33.0 (0.4)	1.59

From the results found in Table 1, the largest increase of Cu and S atomic percentage (at. %) is in the GF/Cu_*x*_S_3 sample, while the lowest is in the GF/Cu_*x*_S_2 sample. To understand the quantities in each synthesized GF/Cu_*x*_S_1, 2, and 3 sample, atomic ratios of Cu/S atoms were calculated. It is seen that the sample with the closest atomic ratio to CuS (1:1) one is GF/Cu_*x*_S_1, while the outermost value is of GF/Cu_*x*_S_2 sample. In conclusion, using different hydrothermal composite synthesis methods yields different Cu/S ratios.

**Figure 3:**
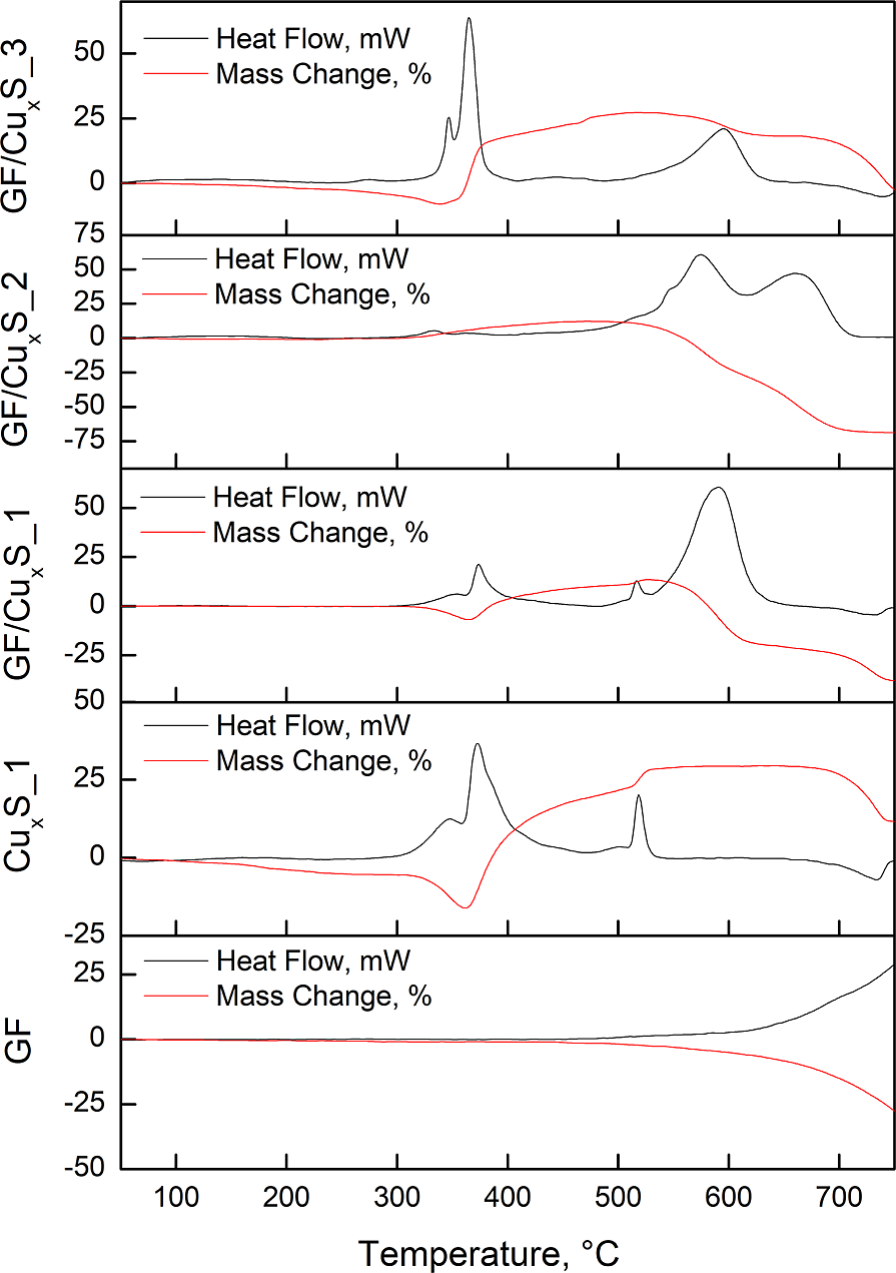
TGA (red) and DSK (black) curves of GF, Cu_*x*_S_1, GF/Cu_*x*_S_1, GF/Cu_*x*_S_2, and GF/Cu_*x*_S_3 samples.

Sample STA was carried out to observe sample mass loss and GF/Cu_*x*_S_1, 2, and 3 thermal stabilities. Thermogravimetry (TGA) and differential scanning colorimetry (DSK) results are listed in Figure 3.

In Figure 3, the TGA curve of the GF sample indicates that the initial sample mass loss is 450 °C, and at 760 °C, the total mass change is equal to 34%. GF sample DSK curve indicated that from the initial mass loss, sample heat flow increased and indicated endothermic energy changes. From Figure 3, it is seen that the initial mass loss of Cu_*x*_S_1 starts at around 300 °C. The analysis was carried out under atmospheric conditions, so after the first initial mass loss, Cu_*x*_S decomposition occurs in four steps:^41^

1. Cu_*x*_S is formed with a lesser amount of sulfur, which leads to the formation of SO_2_
2. Separated copper from Cu_*x*_S starts forming CuO and CuO_2_;
3. Formed copper and sulfur oxides attribute to the formation of oxysulfates: CuO and CuO·CuSO_4_.
4. Formed oxysulfates decompose into CuO.

All of these processes had an influence on sample thermal energy changes, which are of an endothermic and exothermic kind. These Cu_*x*_S decomposition changes are visible in GF/Cu_*x*_S_1 and GF/Cu_*x*_S_3 samples and not much visible in the GF/Cu_*x*_S_2 sample. This sample did not exhibit Cu_*x*_S decomposition curves as observed in the Cu_*x*_S_1 sample and experienced the biggest mass loss, which was equal to −69%. DSK results show energy changes in the decomposition of Cu_*x*_S and all GF/Cu_*x*_S_1, 2, and 3 samples. For samples Cu_*x*_S_1, GF/Cu_*x*_S_1, and GF/Cu_*x*_S_3, from 220 °C up to 650 °C, the decomposition processes were exothermic and had positive heat flow values, while from 650 °C, they became endothermic because of negative heat flow values. The GF/Cu_*x*_S_2 sample in the entire heating temperature range shows no other energy changes other than the exothermic kind. To better understand their thermal energy processes, Cu_*x*_S_1 and all GF/Cu_*x*_S_1, 2, and 3 sample areas below the peaks are considered while calculating sample-specific heat capacity values. These results are presented in Table 2.

**Table 2:** Sample DSK Peak Thermal Heat Capacity Values

sample	temperature interval, °C	specific heat capacity *C*_H_, J/g
Cu_*x*_S_1	350–396	4741.17
	512–527	771.78
	695–745	–722.97
GF/Cu_*x*_S_1	364–389	2406.67
	509–621	10719.06
	702–744	–335.78
GF/Cu_*x*_S_2	313–351	513.91
	526–705	22445.11
GF/Cu_*x*_S_3	257–291	53.58
	354–377	3868.61
	414–479	200.67
	545–626	3417.98
	696–757	–685.71

From the results presented in Table 2, it is seen that all GF/Cu_*x*_S_1, 2, and 3 samples had different endothermic/exothermic reaction temperature intervals and their specific heat capacities *C*_H_ differ. When comparing Cu_*x*_S_1, GF, and GF/Cu_*x*_S_1, 2, and 3 had similar reaction temperature values but different specific heat capacity *C*_H_ values. This means that when Cu_*x*_S composites with GF are synthesized using different hydrothermal methods, they exhibit different thermal properties.

**Figure 4:**
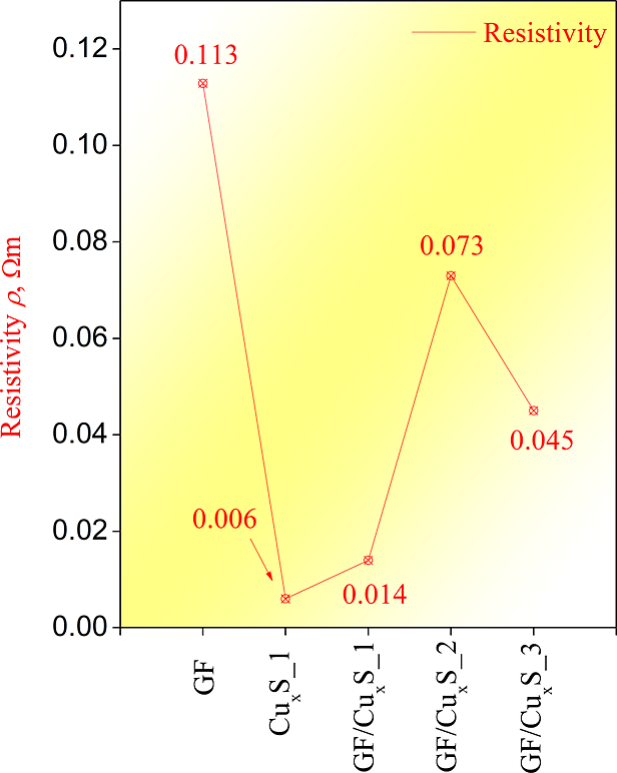
Sample resistivity values.

Sample electrical properties were analyzed using the two-piston method and VA. In this analysis, powdered samples were used for electrical resistance measurements with defined dimensions and under constant pressure. Considering the sample geometric properties, their resistivity ρ values were calculated. Analysis results can be seen in Figure 4.

As observed from sample resistivity measurements, all samples have comparable resistivity ρ values, which differ in the range of several hundredths. GF and Cu_*x*_S_1 samples were also added to the measurements to observe how the use of the hydrothermal method for the Cu_*x*_S_1 synthesis affects its resistivity ρ values. The sample that exhibits the lowest electrical resistivity ρ value was GF/Cu_*x*_S_1, while the sample that exhibits the highest resistivity ρ value was GF. It is important to note that all samples exhibit lower resistivity values when comparing them to GF but higher when comparing them to Cu_*x*_S_1. This means that use of the hydrothermal method for Cu_*x*_S_1 and GF composite synthesis lowers GF resistivity, with the best electrical charge transfer properties exhibited in GF/Cu_*x*_S_1 composite sample ,which has the lowest electrical resistivity ρ value.

**Figure 5:**
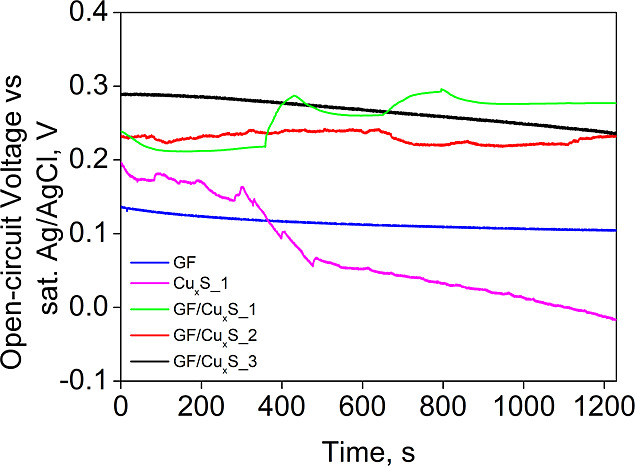
Sample OCV analysis curves.

For electrochemical analysis, OCV and CV methods were used. OCV analysis gives information about equilibrium potential changes at the electrode–electrolyte interface against the reference electrode when no current is flowing through the electrochemical cell. OCV analysis was deployed for sample stabilization before applying CV analysis. GF and Cu_*x*_S_1 samples were also analyzed to compare the prepared composite and raw material properties. These analysis results can be seen in Figure 5.

As observed from the curves shown in Figure 5, all samples, excluding Cu_*x*_S_1 exhibited nonsignificant OCV over time. GF and GF/Cu_*x*_S_3 samples exhibited the best stability out of all the samples with little potential change against the reference electrode during the entire time frame of the experiment. GF/Cu_*x*_S_1 and GF/Cu_*x*_S_2 samples exhibited slight OCV variation, but after around 800 s, they became stable. The Cu_*x*_S_1 sample did not stabilize in the entire time frame of the experiment, with its equilibrium potential against the reference electrode always decreasing to eventually attain negative values. It is also important to mention that the sudden shift of the GF/Cu_*x*_S_1 sample curve could be due to bubble formations on the porous composite electrode surface, while the raw Cu_*x*_S_1 electrode surface was smooth and not porous, which after some time could have expanded as the electrode was submerged in the aqueous electrolyte, which could have led to decreased open-circuit voltage values against the reference electrode.

**Figure 6:**
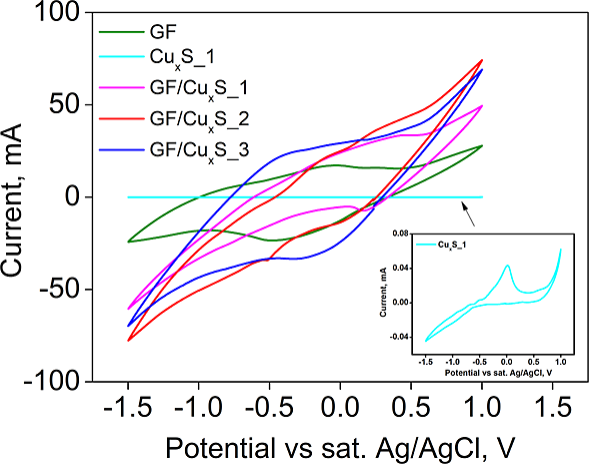
Sample CV analysis curves.

Using CV analysis, sample charge/discharge processes were investigated. These processes are presented as curves, as shown in Figure 6.

From the sample CV curves found in Figure 6, it is seen that the sample curves are observed as similar shapes without any significant redox peaks. These shapes are not necessarily rectangular but show similar current response to the redox reactions occurring on the electrode surface. The sample that exhibits the best current in the potential range of −1.5 to 1.0 V was GF/Cu_*x*_S_2, while GF/Cu_*x*_S_1 and GF/Cu_*x*_S_3 showed a lower current but still higher than that of raw Cu_*x*_S_1 and GF samples. It is important to note that Cu_*x*_S_1 and GF samples as single substances exhibit lower current values than GF/Cu_*x*_S_1, 2, and 3 composites. This could be explained by the electrochemical intercalation and conversion reactions that occur on the electrode matrix. Because of carbon added to the GF/Cu_*x*_S_1, 2, and 3 composites, sodium ions can position themselves into the electrode while having a much larger reaction area. In theory, the electrochemical reaction that takes place on the matrix of the electrode should be a copper sulfide conversion reaction with sodium (5 and 6). These simplified redox reactions are listed down below
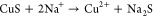
5
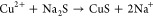
6To analyze how these anode materials match up to others, their areal capacities *C*_s_ were calculated using eq 7 listed below^42^
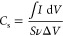
7Here, *C*_s_—areal capacitance of electrode material, mF/cm^2^; *I*—current, mA; *S*—electrode surface area, cm^2^; ν—potential scanning rate, mV/s; Δ*V*—sweep potential window, V. Areal capacities were calculated for anodically and cathodically polarized electrode materials (these values are listed as polarization as cathode/anode and are equivalent to charge/discharge values). Obtained values of capacitance are shown in Table 3.

**Table 3:** Calculated Highest Areal Capacitance Values of Samples

sample	polarization as cathode *C*_cathode_, mF/cm^2^	polarization as anode *C*_anode_, mF/cm^2^
GF	5.45	4.39
Cu_*x*_S_1	0.01	0.01
GF/Cu_*x*_S_1	8.81	10.06
GF/Cu_*x*_S_2	13.65	13.65
GF/Cu_*x*_S_3	12.64	11.49

From the results shown in Table 3, the sample with the highest areal charge/discharge capacitance values of 13.65 mF/cm^2^ was GF/Cu_*x*_S_2. GF/Cu_*x*_S_1 and GF/Cu_*x*_S_3 samples have lower areal charge/discharge capacitance values, which, respectively, are 8.81/10.06 mF/cm^2^ and 12.64/11.49 mF/cm^2^, but these values were still higher than those of Cu_*x*_S_1 and GF sample single substances, which are, respectively, equal to 0.01/0.01 mF/cm^2^ and 5.45/4.39 mF/cm^2^.

## 4 Conclusions

Three different hydrothermal methods of Cu_*x*_S synthesis for GF/Cu_*x*_S_1, 2, and 3 electrode composites have been explored and compared. Using different hydrothermal methods for GF/Cu_*x*_S_1, 2, and 3 sample synthesis leads to different composite structural and morphological properties. When using different GF/Cu_*x*_S_1, 2, and 3 composite preparation methods, sample morphology differs, with small needle-like and flower-like particle structures bound to the GF fibers. Elemental composition of the samples varied with different atomic Cu/S ratios observed in GF/Cu_*x*_S_1, 2, and 3 samples. Thermal analysis results showed that the sample with the largest mass loss and that which showed no endothermic energy changes was GF/Cu_*x*_S_2. The composite sample with the best resistivity value of 0.014 Ω m was GF/Cu_*x*_S_1. As observed from the results of the OCV and CV analyses, the sample that exhibited the best electrical stability and highest charge/discharge areal capacitance values of 13.65/13.65 mF/cm^2^ was GF/Cu_*x*_S_2. In conclusion, the best elemental, morphological, thermal, and electric sample properties were achieved by all GF/Cu_*x*_S_1, 2, and 3 samples, with most of their properties still being better than those of individual Cu_*x*_S_1 and GF raw substances. All used hydrothermal Cu_*x*_S and GF composite synthesis methods yielded good composite properties; therefore, all of these methods are appropriate for Cu_*x*_S and GF composite synthesis in general. When choosing the composite preparation method, it is best to compare their hydrothermal synthesis conditions from which the GF/Cu_*x*_S_1 sample is easiest to prepare. Further studies on these composites are needed to explore their future applications as electrode materials used in SIBs.

## Abbreviations

GF: graphite felt
Cu_*x*_S_1: sample prepared using elemental copper and sulfur
GF/Cu_*x*_S_1: sample prepared using elemental copper; sulfur and graphite felt
GF/Cu_*x*_S_2: sample prepared using copper sulfate pentahydrate; dimethyl sulfoxide and graphite felt
GF/Cu_*x*_S_3: sample prepared using elemental copper; thiourea, dimethyl sulfoxide, ethyl acetate and graphite felt
